# Moving Toward a Near-Total Reduction, Refinement, and Replacement of Live Animal Use in Microvascular Training

**DOI:** 10.7759/cureus.103850

**Published:** 2026-02-18

**Authors:** Danny Kazzazi, Fawz Kazzazi, Georgios Pafitanis

**Affiliations:** 1 Plastic and Reconstructive Surgery, Guy’s and St Thomas’ NHS Foundation Trust, London, GBR; 2 Faculty of Medical Sciences, University College London (UCL), London, GBR; 3 Plastic and Reconstructive Surgery, Royal London Hospital, London, GBR

**Keywords:** animal models, ex vivo, in vivo, medical ethics, microsurgery, plastic surgery training, simulation training, surgical simulation

## Abstract

Simulation training in microsurgery primarily relies on live rat models due to their high-fidelity physiological characteristics, which presents ethical and educational challenges. The increasing emphasis on reducing, replacing, and refining (3Rs) animal use calls for alternative training models that can offer similar educational benefits. We developed a hybrid microvascular training curriculum incorporating non-living models, such as chicken thigh abductor profundus muscle flaps, pork belly intramuscular perforator flap models, and the Micropump for flow-capable microvascular anastomosis training. Trainees progress from basic instrument handling and 2D suturing to more complex microvascular dissections and anastomoses. Skills are evaluated using objective measures such as hand motion analysis, time, economy of movement, and both structural and physiological patency outcomes. The hybrid curriculum has demonstrated a significant reduction in live animal use, estimated up to 80% per trainee (decreasing from approximately 10 rats to just 2 per trainee) while maintaining high educational value. Trainees achieve critical microsurgical competencies through non-living models, with outcomes comparable to traditional live model-based training. Objective assessments show improvements in hand motion, time efficiency, and movement economy, with physiological patency rates closely matching those in live models. This hybrid curriculum addresses both the ethical concerns of animal use and the educational needs of trainees. By incorporating high-fidelity, non-living models and standardized objective assessments, the curriculum provides a viable alternative to live model training. Ongoing validation studies show promising results, suggesting that this curriculum can effectively replace traditional methods without compromising skill acquisition or clinical relevance. Continued refinement of the curriculum is needed to validate its effectiveness against traditional training methods and further reduce live animal usage. The hybrid microvascular training curriculum represents a significant advancement in microsurgical education. By reducing reliance on live animal models and integrating objective skill assessment, it promotes ethical training practices aligned with the 3Rs principles. This curriculum has the potential to revolutionize microsurgical training globally, with future developments aiming to completely replace live animal use.

## Introduction

Simulation training in microsurgery is commonly based on live rat models due to their high-fidelity physiological characteristics [1]. *Microsurgery* refers to surgery performed on small structures, such as blood vessels or nerves, under magnification, using specialized instruments. *Anastomosis* is a core technique involving the suturing of blood vessels to restore continuity of flow, which is assessed by *patency*, a measure of how open and functional the reconnected vessel remains.

However, ethical considerations and the principles of the *3Rs*, i.e., Replacement, Reduction, and Refinement, proposed by Russell and Burch in 1959, have prompted a shift toward non-living models, especially during the early stages of skill acquisition [2]. This transition aligns with the *3Cs*, i.e., Curriculum, Competence, and Clinical performance, framework introduced by Kobayashi et al. in 2015, emphasizing structured training and objective assessments [3]. Globally, many microsurgical training programs are still heavily reliant on live animal models due to their physiological realism, despite increasing ethical, logistical, and financial concerns.

Despite the recognised benefits of non-living models, standardized and universally adopted thresholds for microsurgical competency, particularly those based on objective patency evaluation, remain largely undefined, with existing studies offering variable criteria or focusing on isolated technical endpoints [4,5]. Recent studies have highlighted the need for objective outcome measures to assess curriculum design and establish their relevance to clinical outcomes, thereby supporting the ethical case for limiting animal use while maintaining or improving patient safety [6].

While live animal models are considered the gold standard for microvascular training, non-living models can offer high-fidelity simulations when combined with valid *objective assessment tools*, standardized instruments that evaluate technical performance using measurable outcomes such as time, motion, or anastomotic integrity. These tools not only evidence skill acquisition but also address strict legislation and ethical concerns surrounding animal welfare and patient care [7]. This study utilizes an advanced and refined microvascular training curriculum incorporating non-living, high-fidelity, flow-capable models to reduce the use of live animals in accordance with the 3Rs and 3Cs principles. It underscores the necessity for objective skill assessments to define competency thresholds in microsurgical training [8].

Current microvascular simulation courses often employ ladder-based curricula, allowing trainees to achieve a fairly arbitrary technical competency. However, there is no universally agreed-upon *competency* level to inform ethical training on live animals, as defined by standardized objective assessments clearly related to clinical outcomes. While live animal training offers the advantage of physiological patency assessment, its relevance to clinical performance varies depending on the timing post-anastomosis. Literature reveals controversies over the correlation between performance in live rat courses and clinical outcomes in terms of speed, instrument handling, operative flow, motion, and technical errors. Notably, evaluations of end-product anastomosis patency during such training have not been objectively attempted, which is crucial as a primary outcome [4].

We present the development of a hybrid microvascular training curriculum that optimizes the use of non-living models during the early learning curve. This curriculum employs objective assessments of manual dexterity and outcomes to establish competency thresholds before progressing to live animal model training.

## Technical report

Training model/curriculum

Educational interventions in the hybrid curriculum include 2D and 3D suturing exercises, the use of fresh and cryopreserved biological vessels, two non-living free flap models (the chicken thigh abductor profundus muscle flap and the pork belly intramuscular perforator flap model), and the Micropump, a flow-capable microvascular anastomosis training model.

Microsurgical performance is evaluated through a combination of subjective and objective measures that correlate directly with clinical performance in microvascular anastomosis. These include speed, instrument handling, motion, and procedural flow of operative steps and problem-solving/decision-making.

Objective outcome measures include time, hand motion analysis, and economy of movement, all of which reflect operative fluency and error minimization. In addition, intimal suture line analysis scores are used to assess technical errors, while structural and physiological patency are evaluated using flow-capable models that demonstrate end-product anastomosis outcomes [7].

**Figure 1:**
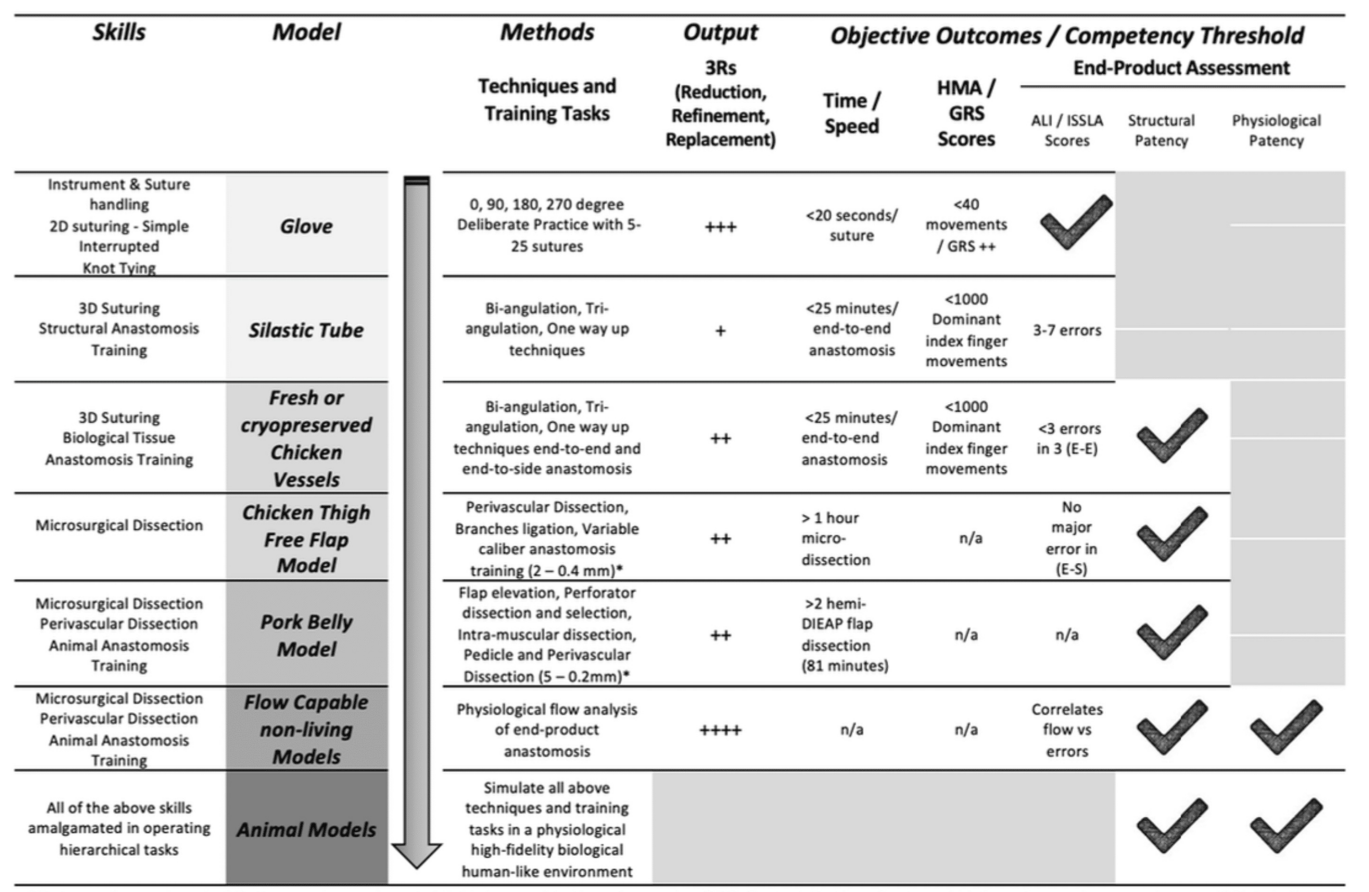
Stepwise training models for the development of microsurgical skills. 2D = two-dimensional; 3D = three-dimensional; HMA = hand motion analysis; GRS = Global Rating Scales; ALI = Anastomosis Lapse Index; ISSLA = Intimal Surface Suture Line Analysis; DIEAP = deep inferior epigastric artery perforator; 'E-E' = end-to-end anastomosis; 'E-S' = end-to-side anastomosis; + = poor; + = moderate; +++ = high; ++++ = very high (impact and enhancement of training according to the ethical considerations of 3Rs) *: Able to reduce the caliber of vessels by using different anatomical regions of chicken thigh and pork belly to train in extremely refined techniques of super-microsurgery, including lymphatics. Image credit: Original figure created entirely by the authors.

The hybrid curriculum stratifies specific skills acquisition and offers an effective progressive step-by-step training model (Figure 1). Once initial instrument handling skills are attained in 2D task practice, anastomosis training is initiated utilizing the structural theory of each anastomotic technique (bi-angulation, triangulation, and back-wall) using traditional non-biological silicone tubes. Early skills in microsurgical dissection are then practiced using biological tissue and chicken thigh free muscle flap models, preparing the trainee for the free rat groin flap in vivo. Deliberate practice using biological vessels in the chicken thigh and the pork belly models allows trainees to develop delicate tissue handling and microvascular dissection techniques.

Progression

Training begins with microscope setup and basic instrument handling using a 2D glove model. Trainees then perform at least 10 end-to-end arterial and venous anastomoses on silicone tubing. Baseline hand motion analysis establishes individual bimanual dexterity/skill in our standardized eight-suture arterial and venous end-to-end anastomosis. The degree of structural patency is assessed after a total of 20 anastomoses are completed by the trainee, utilizing intimal suture analysis scores: Anastomosis Lapse Index (ALI) scores (with a required score of <3 in three consecutive anastomoses), and, in parallel, Intimal Surface Suture Line Assessment (ISSLA) scores to confirm the absence of major errors that would prove fatal in end-to-end and end-to-side anastomosis, respectively.

Hand motion analysis outputs that relate to skill include total path length and the number of hand movements. Our current arbitrary threshold for progression to physiological end-product assessment using the ex vivo flow-capable *Micropump* model is defined as a minimum 50% improvement in skills, specifically hand motion metrics: total path length and number of hand movements, relative to each trainee’s baseline performance. This internally established benchmark, while not yet externally validated, has been adopted within our program as a pragmatic standard to ensure demonstrable skill development before transitioning to flow-based assessment. We are also exploring confirmation of patency with flow measurements and analysis of any non-living model end-to-end anastomosis. Confirmation of structural and physiological patency outcomes with flow would also explore how intimal suture analysis errors affect flow through the anastomosis and provide constructive training feedback (Figure 1).

Findings

Over the past decade, approximately 300 trainees have participated in microsurgical simulation training through our program. The present analysis focuses on 44 plastic surgeons who completed the full hybrid curriculum, comprising 10 novices (no prior microsurgical experience), 24 specialty trainees (experience with 5-50 microvascular anastomoses), and 10 experts (over 100 anastomoses and more than 30 free tissue transfers in the preceding year). All participants progressed through structured stages of the curriculum, culminating in assessment on ex vivo flow-capable models.

Across 44 participants completing the full training pathway, a total of 88 arterial end-to-end anastomoses were analyzed using validated hand motion analysis and end-product assessment tools. Statistically significant differences were demonstrated between experience levels for total time (TT), total movements (TMs), total path length (TP), and ALI score (one-way analysis of variance, all p < 0.0001). Progressive reductions in operative time and error burden were observed from novices to experts, with mean TT decreasing from 3,525 ± 1,067 seconds in novices to 695 ± 232 seconds in experts, and mean ALI score improving from 8.9 ± 1.1 to 2.3 ± 0.9, respectively. Post-hoc Tukey analysis confirmed significant discrimination between most subgroup comparisons, supporting the ability of these objective metrics to differentiate technical proficiency. Learning curve modeling further demonstrated predictable performance plateaus, estimating that approximately 71 anastomoses were required to achieve time-based proficiency and 114 anastomoses to reach error-based patency benchmarks.

These quantitative findings support the progression framework of our curriculum and its ability to benchmark microsurgical skill acquisition before advancing to live model training. Internally defined thresholds, such as a ≥50% improvement in hand motion analysis parameters. are used to determine readiness for flow-based assessment. For generalizability, we note that these outcomes are based on trainees attending simulation courses within the Pan-Thames region, offering a broad representative sample without institutional bias.

Implementation of the hybrid microsurgical training curriculum resulted in a marked reduction in dependence on live animal models without compromising training quality. As a direct result of incorporating non-living models, such as chicken thigh abductor profundus muscle flaps, pork belly intramuscular perforator flap models, and the Micropump for flow-capable microvascular anastomosis, we achieved an estimated reduction in the use of live rats per trainee by up to 80% per trainee, decreasing from approximately 10 rats to just 2 per trainee; equating to 8 animals saved per trainee in a typical training cycle.

The early phases of the curriculum, utilizing ex vivo models, offered notable advantages in foundational skill acquisition. Working with vessels of varying calibers, these models helped trainees develop essential microsurgical and perivascular dissection skills that are directly transferable to real-world clinical scenarios, especially in raising contemporary perforator-based free flaps. These ex vivo training modules replicate the physiological challenges of microsurgery, allowing for more intensive practice and skill development without the immediate need for live animals, supporting a more robust and compliant interpretation of the 3R principles.

As trainees advanced through the curriculum, we saw significant improvements in their proficiency in complex tasks such as microvascular dissections and anastomoses, through the objective measures, hand motion analysis, time efficiency, and economy of movement, which reflected these gains. Structural and physiological patency outcomes achieved in non-living models were comparable to those attained using live animal models, validating the effectiveness of the hybrid curriculum in replicating key learning outcomes of traditional microsurgical training.

**Figure 2:**
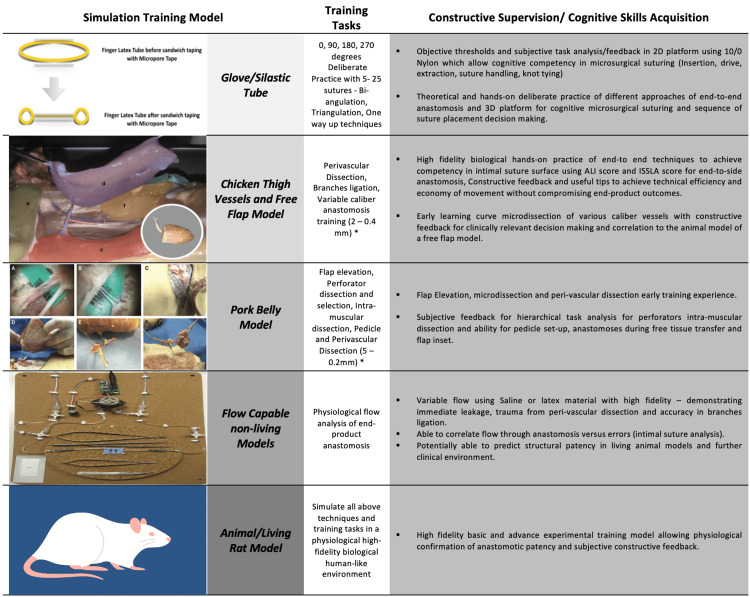
Simulation training models. ALI = Anastomosis Lapse Index; ISLA = Intimal Surface Suture Line Analysis Photographic and schematic summary of simulation models used across the hybrid microsurgical curriculum. The glove and silastic tube schematic is reproduced from Hsieh et al. [9] under CC BY-NC 4.0. The chicken thigh model image is reproduced from Pafitanis et al. [10] under CC BY-NC 4.0. The pork belly dissection image is reproduced from Pafitanis et al. [11] under CC BY-NC 4.0. All remaining images are original photographs taken/created by the authors from the laboratory of Mr Pafitanis. Image credit: Composite created by the authors using a combination of licensed, cited sources and original laboratory photographs.

A visual summary of the simulation models employed, their corresponding training tasks, and the cognitive/technical competencies targeted at each level is presented in Figure 2. This integration highlights how each model contributes to progressive skill acquisition through deliberate practice and structured feedback. The sequence from simple glove-based suturing to more complex models demonstrates a cohesive curriculum aimed at maximizing technical proficiency while minimizing early animal use. The figure also underscores how cognitive decision-making is embedded at each stage, aligning with both 3R principles and competency-based medical education goals.

## Discussion

The implementation of this hybrid training curriculum presents significant potential benefits to a global audience, primarily by enhancing the ethical standards of microsurgical training without compromising educational quality. By relying on high-fidelity non-living models, the curriculum significantly reduces the use of live animals, aligning with global ethical standards and the 3Rs principles [2]. This approach addresses animal welfare concerns while ensuring that surgical trainees acquire necessary skills without ethical compromises.

Recent innovations such as the smartphone-based “noodle technique” described by Ng et al. offer additional low-cost, accessible approaches to early-stage microsurgical training, particularly suited to home environments or resource-limited settings [12]. While these models are valuable for developing basic hand-eye coordination and familiarity with microsurgical instrumentation, they do not replicate the full spectrum of tissue handling, dissection complexity, or flow-based anastomosis assessments incorporated in our hybrid curriculum. Nonetheless, such innovations reflect a global trend toward scalable, ethically conscious microsurgical training solutions and complement more comprehensive curricula such as ours.

The detailed objective assessments used to gauge trainee progress provide standardized measures of competence that can be adopted across various educational settings worldwide, thereby elevating the overall standards of microsurgical education [13]. Tools such as the University of Western Ontario Microsurgical Skills Acquisition/Assessment instrument have demonstrated reliability and validity in objectively assessing microsurgical skills, facilitating consistent evaluation across training programs [5].

Ongoing validation studies help to affirm the efficacy of this curriculum. Benchmarking the hybrid training approach against conventional, live-animal-based models has yielded encouraging results, suggesting that the hybrid curriculum meets, and in some cases exceeds, the skill development benchmarks set by traditional methods [14]. These findings encompass technical outcomes, cost-effectiveness, trainee confidence, and the ability to replicate clinical conditions.

One of the criticisms we must consider is the lack of physiological responses in non-living tissues, which may limit a trainee's ability to develop critical intraoperative judgment under live conditions, such as dealing with vessel spasm, bleeding, or tissue reactivity [15]. Furthermore, some argue that the tactile feedback from synthetic or ex vivo models does not fully replicate the nuances of live tissue handling, which could potentially delay the transferability of skills to the operating theater. While flow-capable models can simulate physiological patency, they still cannot reproduce the dynamic nature of vascular tone and hemodynamics encountered in vivo. In addition, over-reliance on simulated models may risk creating a false sense of proficiency if not carefully benchmarked against validated clinical outcomes. These concerns highlight the importance of ongoing comparative studies and careful integration of live models at later stages to ensure well-rounded surgical competency.

Ultimately, the trajectory of this curriculum points toward the full replacement of live animals in microsurgical training. By continually improving the realism, feedback mechanisms, and clinical relevance of our non-living models, we are working toward a training system that is both ethically responsible and pedagogically sound. This approach could revolutionize microsurgical education, making it more accessible globally while upholding the highest ethical standards in line with the 3R principles.

The next steps in development involve rigorous evaluation and refinement of the hybrid curriculum through ongoing validation studies to ensure its efficacy and relevance are on par with traditional methods that utilize live models. This includes expanding the scope of the curriculum to include more complex microsurgical procedures and integrating emerging technologies that can simulate more realistic surgical environments, such as virtual reality. Furthermore, broadening collaboration with international surgical training programs to adopt this curriculum will promote ethical practices globally. Finally, continued research into improving the fidelity of non-living models and their capacity to mimic real-life surgical challenges will enhance the curriculum’s effectiveness, aiming for a future where the use of live animals in microsurgical training can be minimized or eliminated.

## Conclusions

The development and implementation of a hybrid microvascular training curriculum offers a promising shift toward reducing the reliance on live animals in microsurgical training. By incorporating high-fidelity non-living models and objective skills assessment, this approach supports the ethical principles of 3Rs animal use while maintaining rigorous educational standards. This curriculum provides trainees with a structured, competency-based pathway to develop proficiency before transitioning to living models, thereby optimizing the learning curve and enhancing patient safety. The objective measures embedded in the training process, such as hand motion analysis and patency assessment, facilitate the establishment of standardized competency thresholds, promoting consistency and accountability across training programs globally. As we continue to validate and refine this curriculum, its broader adoption could revolutionize training practices, fostering a more ethical and effective learning environment for future microsurgeons. Expanding this approach through global collaboration and integrating emerging technologies, such as virtual reality, will further improve training outcomes with the potential to ultimately eliminate the necessity for live animal model use in microsurgical training altogether.
